# Estimation of Person Ability under Rapid and Effortful Responding

**DOI:** 10.3390/jintelligence10030067

**Published:** 2022-09-13

**Authors:** Georgios Sideridis, Maisa Alahmadi

**Affiliations:** 1 Boston Children’s Hospital, ICCTR, Harvard Medical School, 300 Longwood Avenue, Boston, MA 02115, USA; 2 Department of Primary Education, National and Kapodistrian University of Athens, Navarinou 13A, 10680 Athens, Greece; 3 Education and Training Evaluation Commission and National Center for Assessment, King Khaled Rd., Riyadh 11534, Saudi Arabia

**Keywords:** rapid responding, effortful responding, item response theory, guessing, carelessness

## Abstract

The goal of the present study was to extend earlier work on the estimation of person theta using maximum likelihood estimation in R by accounting for rapid guessing. This paper provides a modified R function that accommodates person thetas using the Rasch or 2PL models and implements corrections for the presence of rapid guessing or informed guessing behaviors. Initially, a sample of 200 participants was generated using Mplus in order to demonstrate the use of the function with the full sample and a single participant in particular. Subsequently, the function was applied to data from the General Aptitude Test (GAT) and the measurement of cognitive ability. Using a sample of 8500 participants, the present R function was demonstrated. An illustrative example of a single participant, assumed to be either a rapid responder or a successful guesser, is provided using MLE and BME. It was concluded that the present function can contribute to a more valid estimation of person ability.

## 1. Introduction

According to Erwin and Wise (2002), low effort represents the most salient obstacle to accurately estimating a person’s abilities (see also Wise 2015a). This conclusion was drawn based on engaging an “invalid” response vector in the assessment of a person’s abilities, as is the case for random responding and attempts to guess (Wise 2019, 2020a, 2020b) and wandering (Baird et al. 2012; Szpunar et al. 2013). For this reason, several ideas have been put forth aimed at improving the accuracy and validity of personal attributes, some of them focusing on examining the quality aspects of response times (Wise et al. 2020). One such idea was the effort-moderated model put forth by Wise and Demars (2006), in which rapid responding is reflective of disengagement and the display of effort levels that cannot be adequate for an item of a given difficulty level (see also Rios and Soland 2021). Other formulations include the speed–level model (Semmes et al. 2011), the speed–distance model (Fox and Marianti 2016), the lognormal response-time model (RT, van der Linden 2006, 2007), and the 2PL compensatory MIRT model for accuracy (Man et al. 2019). The present study focuses on the Wise and Demars (2006) effort-moderated model, suggesting that a minimum amount of effort is needed to choose the right response among erroneous alternatives for a given test item (Otter et al. 2008); thus, rapid responding is considered detrimental for measurement purposes (Wise 2006, 2009).

Why is it important to incorporate and evaluate response times for our understanding of aptitude and achievement? Several researchers have suggested that fast response times and ability are likely two interchangeable dimensions of the same construct (Kyllonen et al. 1991). We believe that this controversy was solved through factor-analytic studies in which distinct constructs emerged. The role of response time, however, is undoubtedly an important factor in understanding self-regulatory behaviors during test taking (Haladyna 2004). For example, a fast response time, also termed ‘rapid responding’, could indicate superb knowledge and skills or disengagement and effort withdrawal (Rios and Soland 2021, 2022; Soland et al. 2019; Wise 2015b). A slow response time, on the other hand, may be adaptive if a person is careful and thoughtful, thus making good use of the additional time (Foley 2016; San Martín et al. 2006; Ubulom et al. 2012), or maladaptive, as in the case of wandering (Wise 2019; Wise and Kong 2005). Examining response times can provide insights into strategy use and cognitive processing (Jeon and De Boeck 2019) but also aid the valid assessment of individuals, as unpredictable response times can be indicative of aberrant response patterns that invalidate a person’s ability estimation and achievement (Wise et al. 2022). For the above reasons, incorporating response times into our measurement of aptitude can greatly improve our measurement accuracy and validity.

The present study is organized along the following axes. First, item response models are described, including the estimation of up to three parameters, to illustrate random guessing. Second, response behaviors reflective of rapid guessing are defined based on the conceptualizations of Wise and Demars (2006). Third, corrective procedures in the presence of rapid guessing are presented. The paper concludes with the presentation of a modified Baker and Kim (2017) and Rose (2010) function in R using data from a national assessment in Saudi Arabia in which person abilities are estimated using corrective procedures and MLE and BME estimation methods.

## 2. Item Response Models: 2PL and 3PL

The original 2PL model put forth by Birnbaum (1968) is as follows:

(1) $$P\left(Y_{ij}=1|a_{i},\;b_{i},\theta_{j}\right)=\frac{e^{a_{i}\left(\theta_{j}-b_{i}\right)}}{1+e^{a_{i}\left(\theta_{j}-b_{i}\right)}}$$

calculating the probability that person *j* with ability theta (*θ*) is successful on a binary item *i* having a discrimination parameter α*i* and an item difficulty b*ι*. The term e reflects the exponent function. The estimation of person theta is straightforward for the 2PL model but becomes increasingly challenging for the 3PL model, which includes a nonzero lower asymptote, assuming that minus infinity is not the lowest threshold for ability provided that there is some success due to guessing (e.g., random guessing). For example, in multiple-choice tests, a person consistently selecting one option out of five would end up having a success rate of approximately 20% (i.e., 1/5 = 20%) in a balanced test. That estimate would reflect a purely random guessing estimate. Interestingly, as Embretson and Reise (2000) noted, the empirical literature has observed estimates much lower than what was expected from random guessing, most likely because the selection of a response option involves some judgment based on ability. When the ability is low, these judgments may lead to errors exceeding the random guessing (1/m) threshold, while when the ability is high, the likelihood of successfully guessing may increase beyond random guessing. The estimation of the 3PL model has been credited to Birnbaum (1968), assuming a nonzero, low asymptote of the ICC, and is as follows:

(2) $$P\left(Y_{ij}=1|a_{i},\;b_{i},c_{i},\theta_{j}\right)=c_{i}+\left(1-c_{i}\right)\frac{e^{a_{i}\left(\theta_{j}-b_{i}\right)}}{1+e^{a_{i}\left(\theta_{j}-b_{i}\right)}}$$

calculating the probability that person *j* is successful on a binary item *i* given a discrimination parameter *a*, an item difficulty parameter *b*, and an item’s nonzero lower asymptote *c*. The term *e* reflects the exponent function. The 3PL model takes the following form using the item guessing parameter $\gamma_{i}$ in the logistic metric:

(3) $$P\left(Y_{ij}=1|a_{i},\;b_{i},\gamma_{i},\theta_{j}\right)=\frac{e^{\left(\gamma_{i}\right)}}{1+e^{\left(\gamma_{i}\right)}}+\left(1-\frac{e^{\left(\gamma_{i}\right)}}{1+e^{\left(\gamma_{i}\right)}}\right)\frac{e^{a_{i}\left(\theta_{j}-b_{i}\right)}}{1+e^{a_{i}\left(\theta_{j}-b_{i}\right)}}$$

The item guessing parameter c is estimated as follows:

(4) $$c_{i}=\frac{e^{\left(\gamma_{i}\right)}}{1+e^{\left(\gamma_{i}\right)}}$$

with $\gamma_{i}$ being the second threshold of the 3PL model reflecting the lower asymptote of the IRF. When $\gamma_{i}$ is less than −15, the 3PL model reduces to the 2PL model, as the lower asymptote is zero. An extension of the 3PL model is described in detail below, with an attempt to estimate person-based theta by engaging some level of skill in the estimation of guessing, hence introducing person-based guessing (e.g., San Martín et al. 2006).

## 3. Ability-Based Guessing Models

As mentioned above, random guessing is expected when no knowledge or skill is present during the solving process. If this represented a valid argument, then there would be no observations of guessing levels that are at times below what is expected according to chance alone (Embretson and Reise 2000). Several authors have put forth the idea that some degree of successful guessing is a function of a person’s abilities, as more skilled individuals may more easily eliminate erroneous distractors (e.g., in multiple-choice questions—MCQs) compared to less skilled individuals (e.g., San Martín et al. 2006). San Martín et al. (2006) suggested that item-level guessing should be weighted by a person’s abilities using a certain constant term, and this model was slightly extended by Zhu et al. (2019), who stated that: “Correct response probabilities from a solution behavior and guessing behavior increase as the level of ability increases” (p. 449). One goal of the present study was to apply the San Martín idea in the use of ability as a foundation for estimating person-based guessing. Specifically, San Martín et al. (2006) proposed the following model for estimating a person’s likelihood of success on item *i* of latent trait *Y* using a fixed discrimination parameter (excluded for clarity); the model is reduced to a 3PL ability-based guessing model with a fixed discrimination parameter:

(5) $$P\left(Y_{ij}=1|,\;b_{i},\gamma_{i},\theta_{j}\right)=\frac{e^{\left(\theta_{j}-b_{i}\right)}}{1+e^{\left(\theta_{j}-b_{i}\right)}}+\left(1-\frac{e^{\left(\theta_{j}-b_{i}\right)}}{1+e^{\left(\theta_{j}-b_{i}\right)}}\right)*\frac{e^{\left(k\theta_{j}+\gamma_{i}\right)}}{1+e^{\left(k\theta_{j}+\gamma_{i}\right)}}$$

which we supplemented with the inclusion of a discrimination parameter as follows:

(6) $$P\left(Y_{ij}=1|a_{i},\;b_{i},\gamma_{i},\theta_{j}\right)=\frac{e^{a_{i}\left(\theta_{j}-b_{i}\right)}}{1+e^{a_{i}\left(\theta_{j}-b_{i}\right)}}+\left(1-\frac{e^{a_{i}\left(\theta_{j}-b_{i}\right)}}{1+e^{a_{i}\left(\theta_{j}-b_{i}\right)}}\right)*\frac{e^{\left(k\theta_{j}+\gamma_{i}\right)}}{1+e^{\left(k\theta_{j}+\gamma_{i}\right)}}$$

with *k* being a discrimination parameter or slope of the guessing process, so that ability-based guessing incorporates both random guessing and ability-based guessing in the form of a weight defined by parameter *k*. Although values of *k* are defined by the researcher, San Martín et al. suggested a value of .228 as a realistic estimate for their random-guessing example (i.e., ¼ options). The present function allows for the inclusion of a researcher-defined estimate of the slope that combines theta and guessing in the third column of the item parameter file. Thus, in the ability-based model, a person-guessing parameter was introduced contrary to the item-based pseudo-guessing parameter (in Equation (3)), as follows:

(7) $$c_{ij}=\frac{e^{\left(\gamma_{i}+k\theta_{j}\right)}}{1+e^{\left(\gamma_{i}+k\theta_{j}\right)}}$$

which comprises the item-level guessing parameter and weight k to incorporate a person’s ability into the estimation of the person-ability-based guessing index. All the above models involve the estimation of person abilities only; however, as mentioned above, there is ample evidence that time invested in solution-based behaviors is a salient predictor of achievement outcomes. Consequently, the section below describes IRT-based methodologies that consider solution behavior based on response time and ability-based behaviors.

## 4. The Effort-Moderated Model (Wise and Demars 2006)

The original effort-moderated model was put forth by Wise and Demars (2006), who posited that a solution behavior (*SB*) needed to be incorporated into the model so that individuals would be split into rapid and thus non-effortful responders and those displaying solution-based behaviors. Specifically, they suggested that solution behavior could represent a dichotomy of 0–1 based on an overall index of response time effort (*RTE*) estimated as follows:

(8) $$RTE_{j}=\frac{\sum_{i=1}^{k}SB_{ij}}{k}$$

with *k* being the number of items on the instrument under study. They further proposed that the distinction between rapid and effortful responders should involve a judgment based on item-specific response times, with values suggested as indicative of rapid guessing when they reflected individuals responding within the 10th percentile of the response time distribution per item. Further work by Wise and Kong (2005) indicated that the *RTE* measure was internally consistent and exhibited convergent validity with self-reported effort and discriminant validity with SAT scores. Thus, the 3PL effort-moderated model is as follows:

(9) $$P\left(Y_{ij}=1|a_{i},\;b_{i},c_{i},\theta_{j}\right)=\left(SB_{ij}\right)(c_{i}+\left(1-c_{i}\right)\left(\frac{e^{a_{i}\left(\theta_{j}-b_{i}\right)}}{1+e^{a_{i}\left(\theta_{j}-b_{i}\right)}}\right))+\left(1-SB_{ij}\right)\left(g_{i}\right)$$

with *g_i_* reflecting random guessing, i.e., 1/number of options. Given that S*B* only takes values of 0 or 1, rapid guessing would be associated with theta levels equal to random guessing only (*g_i_*), whereas effortful responding would be equivalent to a 3PL model (Equation (2)). Rios and Soland (2021) suggested that a 2PL version of the EM model incorporating *SB* may achieve a higher efficiency by being more parsimonious. Thus, they proposed the following 2PL effort-moderated model:

(10) $$P\left(Y_{ij}=1|a_{i},\;b_{i},\theta_{j}\right)=\left(SB_{ij}\right)\left(\frac{e^{a_{i}\left(\theta_{j}-b_{i}\right)}}{1+e^{a_{i}\left(\theta_{j}-b_{i}\right)}}\right)+\left(1-SB_{ij}\right)\left(g_{i}\right)$$

This also includes random guessing (1/number of options) when rapid guessing is operative.

## 5. Extensions of the Effort-Moderated Model

In the present study, the EM model shown in Equation (10) was implemented along with an extension through which rapid responders obtain a score not only for random guessing but also for informed guessing. This idea is presented by the following conceptualization:

(11) $$P\left(Y_{ij}=1|a_{i},\;b_{i},\theta_{j}\right)=\left(SB_{ij}\right)\left(\frac{e^{a_{i}\left(\theta_{j}-b_{i}\right)}}{1+e^{a_{i}\left(\theta_{j}-b_{i}\right)}}\right)+\left(1-SB_{ij}\right)\left(k\theta_{j}+g_{i}\right)$$

with the last term indicating that rapid guessers obtain a score equal to random guessing *g_i_* plus their estimated theta ability times a slope factor (San Martín et al. 2006). Obviously, the estimated theta ability in Equation (11) originated from the running of a prior model comprising a 2PL model fit to data assuming effortful responding for all individuals. Again, the rationale for (11) lies in the fact that when there is one correct response and several distractors, it is sensible to assume that individuals with a high ability may guess the item correctly, even when their knowledge does not suffice, as they can eliminate one or more erroneous distractors that tap into their relevant skills. Whether high-achieving individuals can eliminate all distractors is irrelevant; if they eliminate more distractors compared to lower-ability individuals, then the odds of success increase significantly to their favor. Thus, Equation (11) extends the Wise and Demars (2006) EM model by incorporating not only rapid by also informed guessing.

## 6. Estimation of Person Theta Using MLE and BME

The two most commonly used analytical derivations of person abilities involve maximum likelihood (ML, Lord 1983; White 1982) and Bayesian estimation (Owen 1975). Both represent important conceptual frameworks, with MLE presenting the limitation that response vectors with no variance (e.g., all zeros or all 1s) cannot be estimated (Bishop et al. 1975), which is why Baker and Kim put forth two conventions for lower and upper theta limits (e.g., theta = ±log(2*length of measure)^1^). On the other hand, BME adds to the estimation of theta the multiplication of the likelihood function with a curve representing some form of prior knowledge. The elaboration of the MLE and BME functions is beyond the scope of the present study. Excellent sources can be found in Rose (2010); Gelman et al. (2003); and others.

## 7. Importance and Goals of the Present Study

This study presents several novelties. First, estimating person theta in the presence of guessing patterns has not yet been investigated, particularly with regard to informed guessing. Third, MLE and Bayesian methodologies were implemented by extending the R function developed by Baker and Kim (2017) and Rose (2010). Consequently, the goal of the present study was to provide MLE and BME estimates of person theta in the presence of random and/or informed guessing. Third, incorporating response times could add stochastic information to the measurement of skills and competencies and provide a more valid estimate of these attributes.

## 8. RBIRT Function in R

The goal of the present R function was to provide MLE and BME estimates of person theta in the presence of random or informed guessing with the application of corrective procedures including the Wise and Demars (2006) random-guessing approach and the San Martín et al. (2006) informed-guessing approach. The function requires as inputs (a) an item parameter matrix that includes item discrimination, item difficulty parameters, and a vector of slopes as per the San Martín model, which is easily retrieved using any commercial or freeware package (e.g., IRTPro, Mplus, R); (b) a response vector with 1s denoting successful responding and 0s failure; and (c) a vector of estimates reflecting rapid or effortful responders. The function returns a list of person theta and standard errors of length equal to those of the input response vector. Specifically, the function is as follows:

RBIRT <- function(model,Nopt,respv,ip,rbe,est)

with each of the function parameters being:

Model: 1 = MLE estimation, 2 = BME estimation;
Nopt: Number of options/scaling as in multiple-choice, Likert-type measures;
Respv: Response vector of person for a given measure;
ip: Item parameter file with the first two columns presenting a and b estimates and the third column the fixed slope defining informed guessing through combining theta with random guessing as per the San Martín et al. (2006) ability-based guessing model;
rbe: A vector defining person rapid (0) versus effortful (1) responding;
est: (1) Rios and Soland model, i.e., random guessing, and (2) San Martín et al. ability-based guessing model;

The present R function is available free of charge through the following GitHub repository: https://github.com/GS1968/CFAtools/blob/main/RBIRT.R (accessed on 6 September 2022).

### 8.1. An Application of the RBIRT Function Using Simulated Data

Response vectors of 200 participants were generated for a seven-item instrument using Mplus 8.8. Item discrimination parameters were estimated to be equal to 1 and item difficulty levels equal to −2, −1, −.5, 0, .5, 1, and 2 logits. The RBIRT function was applied to a single individual (participant 9), who presented the following response vector, with 0 s and 1 s signaling incorrect and correct responses, respectively:
**Item****I1****I2****I3****I4****I5****I6****I7**Person 91101110

Person 9’s theta score according to MLE was equal to 1.015, with a standard error of .927, assuming effortful responding. The respective theta estimate using BME was .995, with an S.E. = .350. If the person was judged not to be effortful, then his/her theta ability was simplified to random guessing (i.e., theta = −1.099) using both MLE and BME based on the Rios and Soland model or −1.322 based on the San Martín model.

### 8.2. An Application of the RBIRT Function in the Measurement of Cognitive Ability

**Figure 1:**
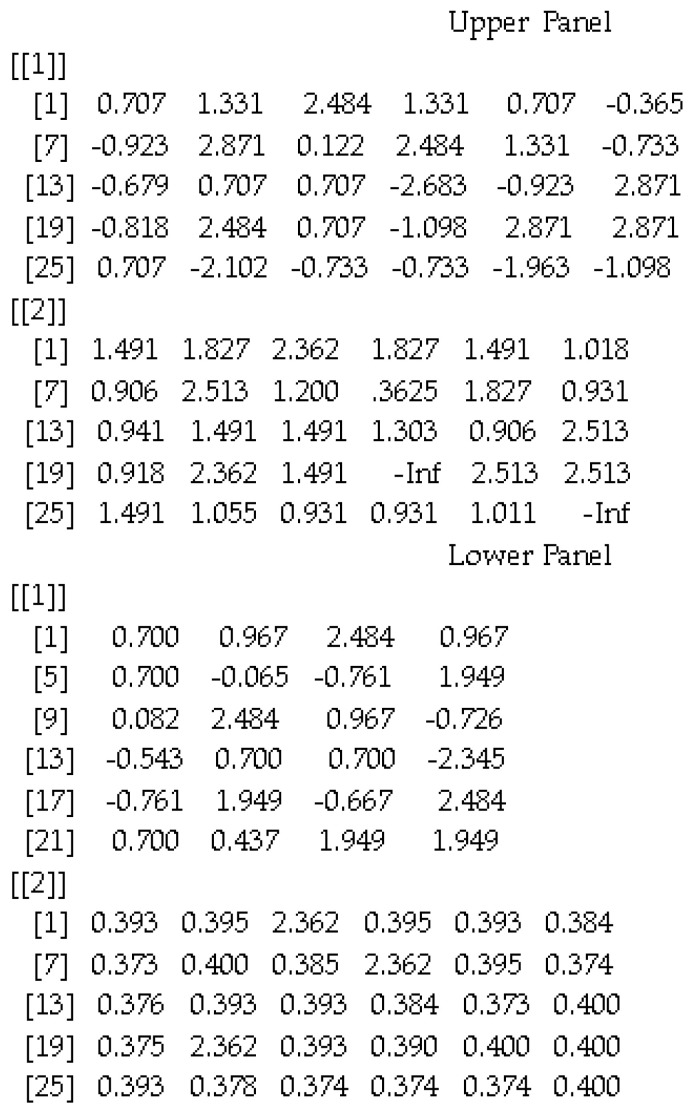
Estimates of theta and standard error for a sample of participants using MLE (upper panel) and BME (lower panel).

The present example involved the measurement of analogy from the computerized version of the General Aptitude Test (GAT), a standardized achievement measure developed and validated in the Kingdom of Saudi Arabia (Dimitrov 2017). Although its function is for the estimation of a single person’s ability, it can be simultaneously applied to a sample of participants. In the present example, theta estimates and standard errors for 8500 individuals were simultaneously estimated. First, the 2PL unidimensional model was determined to be well-fitted to the data according to the Pearson chi-square test (χ^2^(51) = 66.970, *p* = .066), and the root mean square error of approximation (RMSEA) was .01, again indicating exact fit based on the recommendations of MacCallum et al. (1996). Consequently, the obtained item parameter informed the item parameter matrix that was used as the input in the RBIRT function (see Appendix A for model estimation using Mplus 8.8). Further evidence when contrasting the 2PL model and the 2PL EM model was provided by estimating the latter without including rapid responders (Rios and Soland 2021). These results are shown in Table 1 and are indicative of a better model fit for the 2PL EM model compared to the 2PL model, suggesting that the proposition to exclude rapid responders from the estimation process was valid. For the same sample, RBE behaviors were also estimated using Equation (8), suggesting that these individuals exerted 10% less effort compared to the rest of the sample. By applying the RBIRT function to the sample of input parameters listed in Appendix B, we obtained the results shown in Figure 1, with estimates of theta and standard errors of measurement (SEMs).

**Figure 2:**
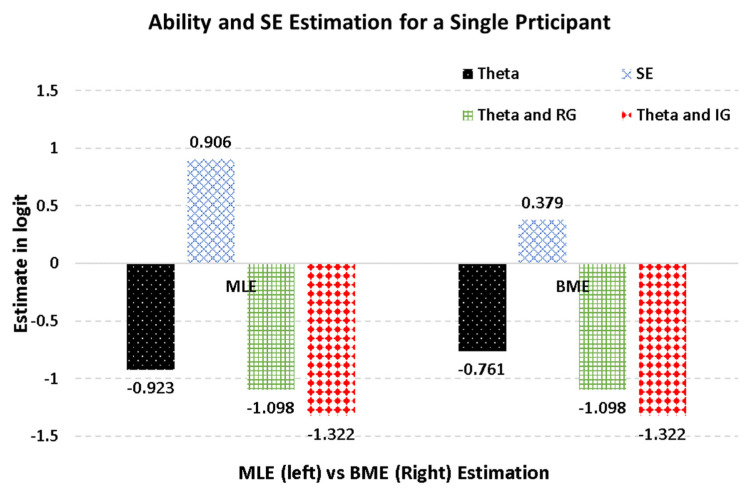
Estimates of an effortful person using MLE, BME, and rapid or effortful responding.

Figure 2 displays the findings for a single individual (case 7) for illustration purposes. Participant 7 presented the following response vector, where 0 reflects an incorrect response and 1 a correct response: 1, 0, 0, 0, 1, 1.

As shown in the figure, the MLE estimate of theta was −.923, with an SE of .906, and the respective BME estimate of theta was −.761, with an SE of .379. These findings agreed with the general premise that SE estimates using BME are generally lower than those using MLE. Case 7 represented an effortful individual, but, if we assume that the person was a rapid responder, his/her theta ability reduced to either random guessing (i.e., −1.098 logit) or ability-based guessing (i.e., −1.322 logits).

## 9. Limitations and Future Directions

The present study is limited for several reasons. First, the goal of the present R function was to estimate person theta, and despite making simultaneous estimations for a sample, the estimations only pertained to a single individual. Second, the estimation process involved the Newton–Raphson algorithm, which is more efficient than the gradient descent algorithm (or the steepest descent), but a plethora of estimators could achieve optimization, each with various pros and cons. Examples are the Bayesian variational inference (VI, Bishop 2006), quasi-Newton procedures, second-order extensions, and alternative direction method multipliers. Furthermore, the corrective procedures adopted herein represent only two of the available recommendations for incorporating person guessing. Other examples include the 2PL model with guessing presented by Zhu et al. (2019), Han’s (2012) fixed guessing approach, and higher-order mixture models (e.g., Lu et al. 2020).

The present study extended the work of Baker and Kim (2017) and Rose (2010) on estimating person theta by using the 2-PL model and applying random guessing and informed guessing recommendations. Furthermore, both MLE and BME approaches were implemented. The current function avoids some of the complexities of the 3PL model by accounting for random or informed guessing within the 2PL framework. This approach preserves the desirable properties of the 2PL logistic model compared to the 3PL model.

## Figures and Tables

**Table 1:** Comparison between 2-PL model and effort-moderated model.

Model	LogLik	Pearson χ^2^/DF	LRT/DF	AIC	BIC	SABIC
2PL	−28709.021	66.976/51	66.169/51	57,442.043	57,526.581	57,488.448
2PL EM	−27070.960	64.389/51	62.742/51	54,165.920	54,249.860	54,211.727

Note. Loglik = Log likelihood; AIC = Akaike information criterion; BIC = Bayesian information criterion; SABIC = sample adjusted BIC.

## Data Availability

Data are available from the second author upon request.

## Note

1 For example, for a 20-item test, lower and upper theta values when response vectors include only 0 s or 1 s would be equal to ±3.68879.

## Appendix A

Annotated Mplus syntax for the estimation of a 2-PL IRT model with dichotomous data.

TITLE: 2PL (IRT) model	! Title of Model to run (label, not required)
DATA: FILE = data.dat;	! Name of data file in fixed asci format
VARIABLE: NAMES = u1-u6;	! Names of items, u1-u6
CATEGORICAL = u1-u6;	! Items are categorical
ANALYSIS: ESTIMATOR = MLR;	! Estimator is Maximum Likelihood with robust errors
MODEL: f BY u1-u6 *;	! One-factor model specified with 6 items, freely estimated
f@1;	! Factor variance fixed to unity for identification
[u1$1-u6$1];	! First threshold estimating item difficulty in logits

## Appendix B

Inputs of R function for Estimating MLE Theta

RBIRT <- function(model,Nopt,respv,ip,rbe,est)

Example:

respv<-read.csv(“ANA2.csv”, sep = “,”, header = FALSE, stringsAsFactors = F)

as.matrix(respv)

ip<-read.csv(“ip.csv”, sep = “,”, header = FALSE, stringsAsFactors = F)

as.matrix(ip)

rbe<-read.csv(“rapid.csv”, sep = “,”, header = FALSE, stringsAsFactors = F)

as.matrix(rbe)

The reader should note that for the estimation of informed guessing (i.e., est = 2), one needs to run the model in two steps. First, run the model with an rbe vector with all 1s so that the theta estimates will be computed regardless of the response speed and will be saved in the folder ‘c:\RBIRT\th.csv’ as a .csv file. Then, the specific theta vector should be saved under the name {thmle} if the model was run with the MLE estimator and {thbme} if the BME estimate was used. These vectors should be read in R or RStudio using the read.csv command, as was carried out for the above vectors (respv, ip, rbe, etc.). Furthermore, the rbe (rapid behavior vector) that includes 0s and 1s should be read again (previously, the model was run with rbe = 1 throughout) so that the estimates of theta under informed guessing can be estimated.

## References

[B1-jintelligence-10-00067] Baird Benjamin, Smallwood Jonathan, Mrazek Michael D., Kam Julia W. Y., Franklin Michael S., Schooler Jonathan W. (2012). Inspired by distraction: Mind wandering facilitates creative incubation. Psychological Science.

[B2-jintelligence-10-00067] Baker Frank B., Kim Seock-Ho (2017). The Basics of Item Response Theory Using R.

[B3-jintelligence-10-00067] Birnbaum A., Lord F. M., Novick M. R. (1968). Some latent trait models and their use in inferring an examinee’s ability. Statistical Theories of Mental Test Scores.

[B4-jintelligence-10-00067] Bishop Christopher M. (2006). Pattern Recognition and Machine Learning.

[B5-jintelligence-10-00067] Bishop Yvonne M., Fienberg Stephen E., Holland Paul W. (1975). Discrete Multivariate Analysis.

[B6-jintelligence-10-00067] Dimitrov Dimiter M. (2017). Examining differential item functioning: IRT-based detection in the framework of confirmatory factor analysis. Measurement and Evaluation in Counseling and Development.

[B7-jintelligence-10-00067] Embretson Susan E., Reise Steven P. (2000). Item Response Theory for Psychologists.

[B8-jintelligence-10-00067] Erwin T. Dary, Wise Steven L., Banta Trudy W. (2002). A scholar-practitioner model for assessment. Building a Scholarship of Assessment.

[B9-jintelligence-10-00067] Foley Brett P. (2016). Getting lucky: How guessing threatens the validity of performance classifications. Practical Assessment, Research, and Evaluation.

[B10-jintelligence-10-00067] Fox Jean-Paul, Marianti Sukaesi (2016). Joint modeling of ability and differential speed using responses and response times. Multivariate Behavioral Research.

[B11-jintelligence-10-00067] Gelman Andrew, Carlin John B., Stern Hal S., Rubin Donald B. (2003). Bayesian Data Analysis.

[B12-jintelligence-10-00067] Haladyna Thomas M. (2004). Developing and Validating Multiple-Choice Items.

[B13-jintelligence-10-00067] Han Kyung T. (2012). Fixing the c parameter in the three-parameter logistic model. Practical Assessment, Research & Evaluation.

[B14-jintelligence-10-00067] Jeon Minjeong, De Boeck Paul (2019). An analysis of an item-response strategy based on knowledge retrieval. Behavior Research Methods.

[B15-jintelligence-10-00067] Kyllonen Patrick C., Tirre William C., Christal Raymond E. (1991). Knowledge and processing speed as determinants of associative learning. Journal of Experimental Psychology: General.

[B16-jintelligence-10-00067] Lord Frederic M. (1983). Maximum likelihood estimation of item response parameters when some responses are omitted. Psychometrika.

[B17-jintelligence-10-00067] Lu Jing, Wang Chun, Zhang Jiwei, Tao Jian (2020). A mixture model for responses and response times with a higher-order ability structure to detect rapid guessing behaviour. British Journal of Mathematical & Statistical Psychology.

[B18-jintelligence-10-00067] MacCallum Robert C., Browne Michael W., Sugawara Hazuki M. (1996). Power analysis and determination of sample size for covariance structure modeling. Psychological Methods.

[B19-jintelligence-10-00067] Man Kaiwen, Harring Jeffrey R., Jiao Hong, Zhan Peida (2019). Joint modeling of compensatory multidimensional item responses and response times. Applied Psychological Measurement.

[B20-jintelligence-10-00067] Otter Thomas, Allenby Greg M., Zandt Trish Van (2008). An integrated model of discrete choice and response time. Journal of Marketing Research.

[B21-jintelligence-10-00067] Owen Roger J. (1975). A Bayesian sequential procedure for quantal response in the context of adaptive mental testing. Journal of the American Statistical Association.

[B22-jintelligence-10-00067] Rios Joseph A., Soland James (2021). Parameter estimation accuracy of the Effort-Moderated Item Response Theory Model under multiple assumption violations. Educational and Psychological Measurement.

[B23-jintelligence-10-00067] Rios Joseph A., Soland James (2022). An investigation of item, examinee, and country correlates of rapid guessing in PISA. International Journal of Testing.

[B24-jintelligence-10-00067] Rose Norman (2010). Maximum Likelihood and Bayes Modal Ability Estimation in Two-Parametric IRT Models: Derivations and Implementation.

[B25-jintelligence-10-00067] San Martín E., Pino Guido del, Boeck Paul De (2006). Irt models for ability-based guessing. Applied Psychological Measurement.

[B26-jintelligence-10-00067] Semmes Robert, Davison Mark L., Close Catherine (2011). Modeling individual differences in numerical reasoning speed as a random effect of response time limits. Applied Psychological Measurement.

[B27-jintelligence-10-00067] Soland James, Wise Steven L., Gao Lingyun (2019). Identifying disengaged survey responses: New evidence using response time metadata. Applied Measurement in Education.

[B28-jintelligence-10-00067] Szpunar Karl K., Moulton Samuel T., Schacter Daniel L. (2013). Mind wandering and education: From the classroom to online learning. Frontiers in Psychology.

[B29-jintelligence-10-00067] Ubulom William J., Amini Clifford M., Island Victoria (2012). Determining the effect of guessing on test scores: An empirical analysis. Scottish Journal of Arts, Social Sciences and Scientific Studies.

[B30-jintelligence-10-00067] van der Linden Wim J. (2006). A lognormal model for response times on test items. Journal of Educational and Behavioral Statistics.

[B31-jintelligence-10-00067] van der Linden Wim J. (2007). A hierarchical framework for modeling speed and accuracy on test items. Psychometrika.

[B32-jintelligence-10-00067] White Halbert (1982). Maximum likelihood estimation of misspecified models. Econometrica.

[B33-jintelligence-10-00067] Wise Steven L. (2006). An investigation of the differential effort received by items on a low-stakes computer-based test. Applied Measurement in Education.

[B34-jintelligence-10-00067] Wise Steven L. (2009). Strategies for managing the problem of unmotivated examinees in low-stakes testing programs. The Journal of General Education.

[B35-jintelligence-10-00067] Wise Steven L. (2015a). Effort analysis: Individual score validation of achievement test data. Applied Measurement in Education.

[B36-jintelligence-10-00067] Wise Steven L. (2015b). Response time as an indicator of test taker speed: Assumptions meet reality. Measurement.

[B37-jintelligence-10-00067] Wise S. L. (2019). An information-based approach to identifying rapid-guessing thresholds. Applied Measurement in Education.

[B38-jintelligence-10-00067] Wise Steven L. (2020a). The impact of test-taking disengagement on item content representation. Applied Measurement in Education.

[B39-jintelligence-10-00067] Wise Steven L. (2020b). Six insights regarding test-taking disengagement. Educational Research and Evaluation.

[B40-jintelligence-10-00067] Wise Steven L., DeMars Christine E. (2006). An application of item response time: The effort-moderated IRT model. Journal of Educational Measurement.

[B41-jintelligence-10-00067] Wise Steven L., Kong Hiaojing (2005). Response time effort: A new measure of examinee motivation in computer-based tests. Applied Measurement in Education.

[B42-jintelligence-10-00067] Wise Steven L., Soland James, Bo Yuanchao (2020). The (non)impact of differential test taker engagement on aggregated scores. International Journal of Testing.

[B43-jintelligence-10-00067] Wise Steven L., Kuhfeld Megan R., Cronin John (2022). Assessment in the time of COVID-19: Understanding patterns of student disengagement during remote Low-Stakes testing. Educational Assessment.

[B44-jintelligence-10-00067] Zhu Zhemin, Wang Chun, Tao Jian (2019). A two-parameter logistic extension model: A n efficient variant of the three-parameter logistic model. Applied Psychological Measurement.

